# Supplementary figures and images for: Periodontal health status in systemic sclerosis patients: Systematic review and meta-analysis
Source: PLoS One. 2024 Feb 2;19(2):e0291078. doi: 10.1371/journal.pone.0291078 (PMC10836703; doi:10.1371/journal.pone.0291078)

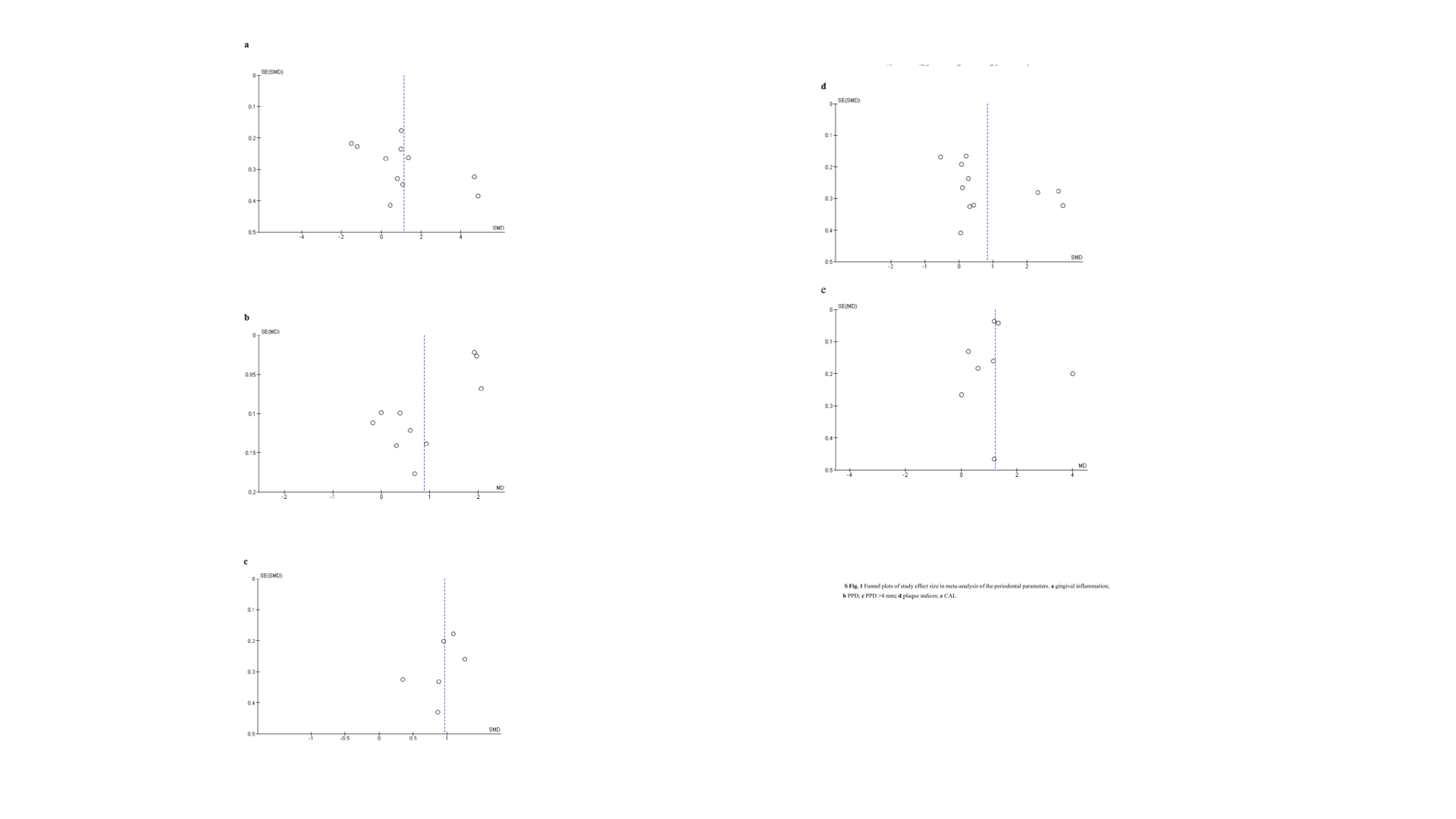

Supplement: S1 Fig — a gingival inflammation; b PPD; c PPD >4 mm; d plaque indices; e CAL. (TIF) [file pone.0291078.s001.tif]
