# Supplementary material for: Periodontal health status in systemic sclerosis patients: Systematic review and meta-analysis
Source: PLoS One. 2024 Feb 2;19(2):e0291078. doi: 10.1371/journal.pone.0291078 (PMC10836703; doi:10.1371/journal.pone.0291078)
Supplement: S2 Table — (DOCX) [file pone.0291078.s003.docx]

Supplemental table 2. Electronic Databases and Search Strategy [February 24, 2023]

| Database (n) | | Search strategy #1 AND #2 |
| --- | --- | --- |
| WoS (n=287)  SCIELO (n=7)  KJD (n=5)  Total (n=295)  <https://www.webofscience.com> | #1 | TS = ((Periodont* OR Gingiv* OR (“Clinical attachment”) NEAR/1 (level OR loss)) OR “Bleeding on probing” OR (Probing NEAR/1 depth$) OR (Plaque AND (index OR score OR presence OR accumulat*)) OR (tooth AND (loss OR mobilit*)) OR Furcation Defects) (n=176,076) |
|  | #2 | TS = ((systemic NEAR/1 sclerosis) OR Scleroderma) (n=38,229) |
| Scopus  (n=176)  <https://www.scopus.com> | #1 | TITLE-ABS-KEY ((Periodont* OR Gingiv* OR (“Clinical attachment”) W/1 (level OR loss)) OR “Bleeding on probing” OR (Probing W/1 depth$) OR (Plaque AND (index OR score OR presence OR accumulat*)) OR (tooth AND (loss OR mobilit*)) OR “Furcation Defects”) (n=114,634) |
|  | #2 | TITLE-ABS-KEY ((systemic W/1 sclerosis) OR Scleroderma) (n=46,267) |
| PubMed  (n=137)  <https://pubmed.ncbi.nlm.nih.gov/> | #1 | "Periodontal Diseases"[Mesh] OR "Periodontitis"[Mesh] OR "DMF Index"[Mesh] OR (Periodont*[Title/Abstract] OR Gingiv*[Title/Abstract] OR ("Clinical attachment"[Title/Abstract]) AND (level[Title/Abstract] OR loss[Title/Abstract])) OR "Bleeding on probing"[Title/Abstract] OR (Probing[Title/Abstract] AND depth[Title/Abstract]) OR (Plaque[Title/Abstract] AND (index[Title/Abstract] OR score[Title/Abstract] OR presence[Title/Abstract] OR accumulat*[Title/Abstract])) OR (tooth[Title/Abstract] AND (loss[Title/Abstract] OR mobilit*[Title/Abstract])) OR "Furcation Defects"[Title/Abstract] (n=147,259) |
|  | #2 | "Scleroderma, Systemic"[Mesh] OR "systemic sclerosis"[Title/Abstract] OR "Scleroderma"[Title/Abstract] (n=32,263) |
|  |  |  |
|  |  |  |
| CENTRAL  (n=10)  <https://www.cochranelibrary.com/> | #1 | [mh “Periodontal Diseases”] OR [mh “Periodontitis”] OR [mh “DMF Index”] OR (Periodont* OR Gingiv* OR (“Clinical attachment”) NEAR/1 (level OR loss)) OR “Bleeding on probing” OR (Probing NEAR/1 depth$) OR (Plaque AND (index OR score OR presence OR accumulat*)) OR (tooth AND (loss OR mobilit*)) OR Furcation Defects (n=29,220) |
|  | #2 | [mh “Scleroderma, Systemic”] OR (systemic NEAR/1 sclerosis) OR Scleroderma (n=1,900) |
| n - number of hits, WoS - Web of Science Core Collection, SCIELO - SciELO Citation Index, KJD - Korean Journal Database, CENTRAL - Cochrane Central Register of Controlled Trials, TS - Topic (article title, abstract and keywords) | | |
