# Supplementary material for: Periodontal health status in systemic sclerosis patients: Systematic review and meta-analysis
Source: PLoS One. 2024 Feb 2;19(2):e0291078. doi: 10.1371/journal.pone.0291078 (PMC10836703; doi:10.1371/journal.pone.0291078)
Supplement: S3 Table — A—Inappropriate study design; B—Insufficient data. (DOCX) [file pone.0291078.s004.docx]

Supplemental table 3. The study excluded during full-text screening

| Studies | Reasons for exclusion |
| --- | --- |
| Yuen HK, Weng Y, Reed SG, Summerlin LM, Silver RM. Factors associated with gingival inflammation among adults with systemic sclerosis. Int J Dent Hyg. 2014 Feb;12(1):55–61. | B |
| Scardina GA, Pizzigatti ME, Messina P. Periodontal microcirculatory abnormalities in patients with systemic sclerosis. J Periodontol. 2005 Nov;76(11):1991–5. | B |
| Ozcelik O, Haytac MC, Ergin M, Antmen B, Seydaoglu G. The immunohistochemical analysis of vascular endothelial growth factors A and C and microvessel density in gingival tissues of systemic sclerosis patients: their possible effects on gingival inflammation. Oral Surg Oral Med Oral Pathol Oral Radiol Endod. 2008 Apr;105(4):481–5. | A |
| Ancuta C, Pomirleanu C, Iordache C, Fatu A, Popescu E, Ancuta E, et al. Periodontal Disease and Lipid Profile in Systemic Sclerosis: an EUSTAR Cohort Experience. Rev Chim. 2017 Apr;68(4):890–3. | A |
| Ozcan M, Ciftci V, Turk I. The relationship between oral and periodontal findings with disease type in scleroderma patients. CUKUROVA Med J. 2021;46(1):39–45. | A |
| Baron M, Hudson M, Dagenais M, Macdonald D, Gyger G, El Sayegh T, et al. Relationship Between Disease Characteristics and Oral Radiologic Findings in Systemic Sclerosis: Results From a Canadian Oral Health Study. Arthritis Care Res. 2016 May;68(5):673–80. | B |
| Zalewska A, Knaś M, Gińdzieńska-Sieśkiewicz E, Waszkiewicz N, Klimiuk A, Litwin K, et al. Salivary antioxidants in patients with systemic sclerosis. J Oral Pathol Med. 2014 Jan;43(1):61–8. | A |
| Thum-Tyzo, K.; Balawejder, A., et al. Occurrence of oral lesions in systemic sclerosis: Dent. Med. Probl. 2010; 47, (1):53-60. | B |
| Poole J, Conte C, Brewer C, Good CC, Perella D, Rossie KM, et al. Oral hygiene in scleroderma: The effectiveness of a multi-disciplinary intervention program. Disabil Rehabil. 2010;32(5):379–84. | A |
| Yang KT, Wei JCC, Chang R, Lin CC, Chen HH. Association between Appendicitis and Incident Systemic Sclerosis. J Clin Med. 2021 May 27;10(11). | A |
| Yuen HK, Weng Y, Bandyopadhyay D, Reed SG, Leite RS, Silver RM. Effect of a multi-faceted intervention on gingival health among adults with systemic sclerosis. Clin Exp Rheumatol. 2011 Apr;29(2 Suppl 65):S26-32. | A |

A - Inappropriate study design; B - Insufficient data
