# Supplementary material for: Periodontal health status in systemic sclerosis patients: Systematic review and meta-analysis
Source: PLoS One. 2024 Feb 2;19(2):e0291078. doi: 10.1371/journal.pone.0291078 (PMC10836703; doi:10.1371/journal.pone.0291078)
Supplement: S4 Table — *CD, cannot determine; NA, not applicable; NR, not reported. (DOCX) [file pone.0291078.s005.docx]

Supplemental table 4. Quality assessment of included studies

| Study NHLBI checklist for | Wood 1988 | Chu 2011 | Leung 2011 | Matarese 2012 | Mayer 2013 | Baron 2014 | Elimelech 2015 | Pischon 2016 | Isola 2017 | Iordache 2019 | Da Silva 2019 | Polizzi 2020 | Isola 2021 | Buchbender 2021 |
| --- | --- | --- | --- | --- | --- | --- | --- | --- | --- | --- | --- | --- | --- | --- |
| Was the research question or objective in this paper clearly stated and appropriate? | YES | YES | YES | YES | YES | YES | YES | YES | YES | YES | YES | YES | YES | YES |
| Was the study population clearly specified and defined? | YES | YES | YES | YES | YES | YES | YES | YES | YES | YES | YES | YES | YES | YES |
| Did the authors include a sample size justification? | NO | NO | NO | NO | NO | NO | NO | NO | NO | NO | NO | YES | NO | NO |
| Were controls selected or recruited from the same or similar population that gave rise to the cases? | YES | YES | YES | YES | YES | YES | YES | YES | YES | YES | YES | YES | YES | YES |
| Were the definitions, inclusion and exclusion criteria, algorithms or processes used to identify or select cases and controls valid, reliable, and implemented consistently across all study participants? | NR | NR | YES | YES | YES | YES | YES | YES | YES | YES | YES | YES | YES | YES |
| Were the cases clearly defined and differentiated from the controls? | YES | YES | YES | YES | YES | YES | YES | YES | YES | YES | YES | YES | YES | YES |
| If less than 100 percent of eligible cases and/or controls were selected for the study, were the cases and/or controls randomly selected from those eligible? | CD | CD | CD | CD | CD | CD | CD | CD | CD | CD | CD | CD | CD | CD |
| Was there use of concurrent controls? | CD | CD | CD | CD | CD | CD | CD | CD | CD | CD | CD | CD | CD | CD |
| Were the measures of exposure/risk clearly defined, valid, reliable, and implemented consistently (including the same time) across all study participants? | YES | YES | YES | YES | YES | YES | YES | YES | YES | YES | YES | YES | YES | YES |
| Were the assessors of exposure/risk blinded to the case or control status of participants? | CD | CD | CD | CD | CD | CD | CD | CD | CD | CD | CD | CD | CD | CD |
| Were key potential confounding variables measured and adjusted statistically in the analyses? If matching was used, did the investigators account for matching during study analysis? | NO | YES | YES | YES | YES | YES | NO | YES | YES | YES | NO | YES | YES | YES |
| Quality rating | Fair | Fair | Good | Good | Good | Good | Fair | Good | Good | Good | Fair | Good | Good | Good |

*CD, cannot determine; NA, not applicable; NR, not reported
