# Supplementary material for: Periodontal health status in systemic sclerosis patients: Systematic review and meta-analysis
Source: PLoS One. 2024 Feb 2;19(2):e0291078. doi: 10.1371/journal.pone.0291078 (PMC10836703; doi:10.1371/journal.pone.0291078)
Supplement: Attachment — Submitted filename: Response to Reviewers.docx [file pone.0291078.s006.docx]

**Stefano Corbella, DDS, PhD
Academic Editor
PLOS ONE**

**AUTHOR'S POINT-BY-POINT RESPONSE TO REVIEWERS**

Thank you for allowing us to submit a revised version of our manuscript titled **"Periodontal health status in systemic sclerosis patients: systematic review and meta-analysis" (PONE-D-23-12300) to *PLOS ONE.* We are grateful for the valuable comments provided by the reviewers. We have taken great care to incorporate the suggested changes in order to address the reviewers' recommendations. The revised portions of the manuscript are highlighted in yellow, and detailed explanations of the modifications have been provided in our responses.**

**Academic Editor**

GENERAL COMMENTS

Thank you for submitting your manuscript to PLOS ONE. After careful consideration, we feel that it has merit but does not fully meet PLOS ONE’s publication criteria as it currently stands. Therefore, we invite you to submit a revised version of the manuscript that addresses the points raised during the review process.

**Response:** Dear Academic Editor, we would like to express our gratitude for providing us with the opportunity to enhance our manuscript and resubmit it to *PLOS ONE*. We have taken the reviewers' comments and suggestions into careful consideration and have made thorough revisions accordingly. To the best of our knowledge, this systematic review and the meta-analysis, represent the first comprehensive analysis focused exclusively on the periodontal status of patients with systemic sclerosis. We are hopeful that the manuscript has been significantly improved and that it now meets the standards for publication in *PLOS ONE*.

Reviewer #1:

1**. Please edit language throughout the text where needed.**

**Response:** We would like to thank the reviewer for this comment. A thorough English language check has been conducted by Irena Aleksic-Hajdukovic, Assistant Professor in English Language at the School of Dental Medicine, University of Belgrade to ensure the accuracy and clarity of the manuscript.

**2. Reg your PICO: impaired periodontal condition is not properly articulated, perhaps you need to rephrase to worse instead of impaired.**

**Response:** In accordance with the Reviewer’s suggestion, we have rephrased the PICO question: “Do systemic sclerosis patients have worse periodontal health in comparison with non-systemic sclerosis individuals?”.

The amended sentence in the manuscript is highlighted in yellow.

**3. Abbreviations of your main outcomes do not align with the outcomes, FMPS instead of PI and also references are missing.**

**Response:** We would like to thank the Reviewer for noticing this omission. We used the abbreviation “PI” in the manuscript and made the correction in the main outcomes.

“The references of the PI used in the included studies are listed in the section **Descriptive results of the included studies**: Eight of the eleven studies evaluating the presence of plaque used the PI according to Silness and Loe [1–8], two studies used the dichotomous plaque score [9,10], and one used the Mombelli Plaque Index [11].”

This sentence is highlighted in yellow in the manuscript.

**4. The agreement between the independent reviewers is not reported in your results.**

**Response:** We would like to thank the Reviewer for pointing this out. In order to clarify this issue, we have added and highlighted in yellow the following sentences in the **Results** section:

“Owing to the a priori-defined eligibility criteria, there were no disagreements between the independent reviewers in the process of article selection and inclusion. Additionally, a very high percentage of agreement (i.e., 95%) between the independent reviewers was observed during the process of quality assessment of the articles included in the final review. Furthermore, any disagreements were resolved by another author (N.N. J).”

**5. Your review cannot be both narrative and systematic, you may decide what it is and use the term consistently.**

**Response:** We would like to thank the Reviewer for noticing this inconsistency. We made the correction and adhered to the term *systematic*.

**6. How did you calculate the missing means before pooling outcomes?**

**Response:** We would like to thank the Reviewer for this question. Chapter 6, Section 6.5.2.9 of the Cochrane Reviewers’ Handbook [1] suggests the utilization of statistical methods proposed by Wan et al. [2] and Bland [3] for imputing a missing mean value based on the median. In order to enhance the clarity of the article, we have included additional explanations in the text: “When the data for the same outcome were presented differently (e.g., the mean in one study, the median in another), the statistical approaches recommended by the Cochrane Reviewers’ Handbook were used to convert the medians into means to allow data combining [22–24].”

This sentence and the references added are highlighted in yellow in the manuscript.

[1] Higgins JPT, Li T, Deeks JJ (editors). Chapter 6: Choosing effect measures and computing estimates of effect. In: Higgins JPT, Thomas J, Chandler J, Cumpston M, Li T, Page MJ, Welch VA (editors). Cochrane Handbook for Systematic Reviews of Interventions version 6.3 (updated February 2022). Cochrane, 2022. Available from www.training.cochrane.org/handbook., n.d.

[2] Wan X, Wang W, Liu J, Tong T. Estimating the sample mean and standard deviation from the sample size, median, range and/or interquartile range. BMC Med Res Methodol 2014;14:135. <https://doi.org/10.1186/1471-2288-14-135>.

[3] Bland M. Estimating Mean and Standard Deviation from the Sample Size, Three Quartiles, Minimum, and Maximum. Int J Stat Med Res 2014;4:57–64. https://doi.org/10.6000/1929-6029.2015.04.01.6.

**7. Was the clinical measurements comparable across studies? Probes? Clinicians? Specialists? Private practice/uni? Calibrations?**

**Response:** We would like to thank the Reviewer for pointing this out. We have added a few sentences in the **Results** section to provide further clarification. These sentences are highlighted in yellow in the manuscript:

“All the included studies utilized manual periodontal probes for conducting periodontal examinations. Specifically, the PCP 15 periodontal probe (Hu-Friedy, Chicago, Illinois, USA) was predominantly used, i.e., in six studies [11,13,29,30,32], three studies employed the Community Periodontal Index (CPI) probe [14,15,27], and one study each used the Williams PCP 2 [16], the PCP 11[12] and the PCP 12 [33] probes. The specific type of manual probe used in the remaining studies was not reported. In all studies, the clinical examiners were described as experienced. Examiner calibration was performed in the majority of the included studies [13,16,25,28–31,33] to ensure consistency and accuracy of the assessment.”

**8. You mention that the etiology of perio in SS is multifactorial (?) which is not very accurate. This is true for periodontitis in general. Also SS is one variant of sclerosis which is important for the reader to know along with some more information on the condition.**

**Response:** We would like to thank the Reviewer for bringing this to our attention. We have expanded the **Introduction** section to provide better clarification and more information on systemic sclerosis. The sentences added are highlighted in yellow in the manuscript:

“SSc has been reported to be three times more prevalent in females than in males, with the frequency peaks in the sixth and seventh decades of life [2]. This systemic disease is associated with higher mortality rates compared to other rheumatic diseases with a five-year cumulative survival rate of 75% [3]. The hallmark of SSc is microvascular damage which triggers immune cells to produce autoantibodies as well as proinflammatory and profibrotic cytokines. This cascade of events leads to excessive collagen production by means of activated fibroblasts and myofibroblasts, thus resulting in an accumulation of collagen in the skin and internal organs [4].

Apart from the known factors that may contribute to periodontal destruction in SSc patients, such as increased plaque acummulation, it is likely that there are additional underlying mechanisms involved and these require further examinations [16].

**9. I would like to see discussion on the potential direction of the etiological link--in my view it would be more likely that the SS can burden periodontal status for practical but**

**Response:** One paragraph in the **Discussion** section is related to the potential direction of the etiological link (i.e., lines 362-372). According to the data available from the included studies we concluded the disccussion on this topic in the following way: ”Although the link between the two diseases has not yet been fully established, studies suggest that poor oral hygiene, proinflammatory cytokines, and microvascular alterations in SSc patients may promote the onset and progression of periodontitis.“ In addition, we ended the **Discussion** section by stating the following: „The relationship between SSc and periodontitis should be investigated further since periodontitis represents a known risk factor for low-grade systemic inflammation and some systemic diseases [46,47].“. Also, in the **Conclusion** section we reiterate this:” Further research with a higher level of evidence is needed to clarify the relationship between the two diseases and establish protocols and recommendations for periodontal treatment of SSc patients.”

These sentences are highlighted in yellow in the manuscript.

**10. Generally, I would phrase very carefully and tone down the possible link in view of the very sample sizes of the included studies.**

**Response:** We agree with the Reviewer. We discussed the small sample size of some of the included studies and presented this as a limitation of this systematic review. Additionally, we pointed out that all included studies used case-control designs, therefore, the cause-effect relationship between SSc and periodontitis could not be fully elucidated. We ended the **Conclusion** section with claiming that: “Future research with a higher level of evidence is needed to clarify the relationship between the two diseases and establish protocols and recommendations for periodontal treatment of SSc patients.”

These sentences are highlighted in yellow in the manuscript.

**Reviewer #2:** Interesting and well executed study, although it exhibits the inherent problems when a meta-analysis includes observational studies. Only a minor comment:
- Please perform a thorough english language check as many typo and grammatical errors exist.

**Response:** We would like to thank the Reviewer for these supportive and encouraging words. A thorough English language check has been conducted by Irena Aleksic-Hajdukovic, Assistant Professor in English Language at the School of Dental Medicine, University of Belgrade, to ensure the accuracy and clarity of the manuscript.
